# A Simple Genotyping Method for Rapid Differentiation of *Blastocystis* Subtypes and Subtype Distribution of *Blastocystis* spp. in Thailand

**DOI:** 10.3390/pathogens8010038

**Published:** 2019-03-21

**Authors:** Nittaya Srichaipon, Surang Nuchprayoon, Sarit Charuchaibovorn, Pattadon Sukkapan, Vivornpun Sanprasert

**Affiliations:** 1 Lymphatic Filariasis and Tropical Medicine Research Unit, Chulalongkorn Medical Research Center, Faculty of Medicine, Chulalongkorn University, Bangkok 10330, Thailand; nttychai@gmail.com (N.S.); fmedstt@gmail.com (S.N.); sarit.src@gmail.com (S.C.); 2 Department of Parasitology, Faculty of Medicine, Chulalongkorn University, Bangkok 10330, Thailand; 3 Program of Biology, Faculty of Science, Chandrakasem Rajabhat University, Bangkok 10900, Thailand; pattadon.s@chandra.ac.th

**Keywords:** *Blastocystis* spp., subtypes, subtyping method, subtype distribution, Thailand

## Abstract

*Blastocystis* spp. is one of the most common protozoa of humans and animals worldwide. The genetic diversity of *Blastocystis* spp. might be associated with a wide range of symptoms. However, the prevalence of each subtype is different in each country. Until now, there is no standard method for subtyping of *Blastocystis* spp. We developed a sequential restriction fragment length polymorphism (RFLP) analysis for the rapid differentiation of human *Blastocystis* subtypes. A large-scale study was also conducted to determine the subtype distribution of *Blastocystis* spp. in Thailand. Stool samples were collected from 1025 school-age students in four regions of Thailand. *Blastocystis* infections were identified by direct smear, formalin ethyl-acetate concentration technique (FECT), Boeck and Drbohlav’s Locke-Egg-Serum (LES) medium culture, and polymerase chain reaction (PCR) of small-subunit ribosomal DNA (SSU rDNA). Subtypes of *Blastocystis* spp. were determined by RFLP. Phylogenetic tree of partial SSU rDNA sequences of *Blastocystis* spp. was constructed using the Maximum Likelihood (ML) method. Out of 1025 students, 416 (40.6%) were positive for *Blastocystis* spp. Using two steps of RFLP reactions, we could determine subtype one–three among these students. Subtype 3 was the most common subtype (58.72%) in Thai students, followed by subtype 1 (31.2%), and subtype 2 (10.1%). *Blastocystis* subtype 3 was the most prevalent in all regions of Thailand. The subtype distribution of *Blastocystis* spp. in Thailand was different from other countries.

## 1. Introduction

*Blastocystis* spp. is a common enteric protozoan in human and animals worldwide. Little is currently known about the biology of *Blastocystis* spp. Until now, taxonomy, life cycle, mode of transmission, as well as treatment management of the *Blastocystis* infection remains unclear. Moreover, conflicting studies or reports concerning the pathogenicity of the protozoan continue to be published. In the past, *Blastocystis* spp. was thought to be pathogenic only in immunocompromised patients [1]. The public health significance of *Blastocystis* infection has increased in the last decade. *Blastocystis* infection causes various symptoms in immunocompetent individuals in the absence of any parasitic co-infection [2,3]. Prevalence of *Blastocystis* infection is about 1%–23% in developed countries [4,5], and 10%–100% in developing countries [6,7,8]. In Thailand, the prevalence of *Blastocystis* infection is 10%–45% [9,10,11,12,13].

*Blastocystis* infection can cause a wide range of clinical manifestations. Although most of the infected people are asymptomatic, some individuals show gastrointestinal symptoms, such as abdominal pain, acute diarrhea, chronic diarrhea, constipation, flatulence, nausea, vomiting, thirst, insomnia, anorexia, and weight loss [14]. Dermatological symptoms (e.g., rash, itching, and urticaria) in *Blastocystis* infected people have also been reported [15,16,17]. However, the cause of different clinical symptoms are still unknown. It might be due to the morphology diversity [17,18], genetic diversity of protozoa [19], or different host’s immune responses [20].

Molecular techniques have revealed extensive genetic diversity of *Blastocystis* spp. [21]. Until now, about 17 subtypes have been reported [22]. Subtype 1–9 have been reported in humans, animals, mammals, and birds [23,24]. Subtype 10 has been isolated from cattle, sheep, deer [23], subtype 14 has been isolated from cattle [25], and subtype 11–16 have been isolated from elephant, giraffe, and quokka in the zoo [26]. Recently, subtype 17 has been isolated from gundi [22]. The major subtypes (about 90%) found in humans are subtype 1–4 [22]. However, it is possible that some subtypes of *Blastocystis* spp. can cross the host species barriers and cause zoonotic transmission [27]. The genetic diversity of the protozoa has led to the suggestion that the subtypes of *Blastocystis* spp. might be associated with a wide range of clinical symptoms. Genotyping of *Blastocystis* spp. between the asymptomatic and symptomatic isolates might be useful in determining the pathogenicity of the protozoa. Subtype distribution of *Blastocystis* spp. considerably varies within and between countries and regions in each country [12,28]. Molecular epidemiological studies of *Blastocystis* infection have reported the different prevalence of subtypes from symptomatic and asymptomatic patients in several countries [3,29,30,31,32]. However, the association between subtypes of *Blastocystis* spp. and the clinical symptoms remain controversial. 

Although there are many molecular methods for analyzing subtypes of *Blastocystis*, most techniques need to be performed by several reactions to determine the subtype of the protozoan. The most common technique is PCR with subtype-specific primers [19,33,34,35]. However, only seven primer sets for subtype 1–7 have been reported [33,34]. The PCR with specific primers also shows moderate sensitivity because some isolates are not detected with any of these primers [34,36]. Moreover, this method is not appropriate for the detection of mixed subtype infection [37]. Another common technique for genotyping of *Blastocystis* spp. is PCR together with DNA sequencing [24,35,38,39]. However, sizes of PCR amplicons are usually longer than 1000 bp, resulting in the difficulty to amplify, and the cost of DNA sequencing. Nested PCR of SSU rDNA has also been developed to detect *Blastocystis* protozoa directly from stool samples with the higher sensitivity [40,41], but it is still necessary to perform DNA sequencing to identify the subtypes [11]. PCR-RFLP of SSU rDNA has also been used for genetic variation analysis of *Blastocystis* spp. showing the different RFLP patterns of various isolates of *Blastocystis* spp. [42,43,44,45,46]. However, only some studies report PCR-RFLP analysis with different primers and restriction enzymes to identify subtypes of *Blastocystis* spp. [44,45,46]. Moreover, after using three restriction enzymes in RFLP analysis, *Blastocystis* will be categorized into four groups after that PCR with specific primers have to be performed to identify the subtype of the parasites [46]. 

In 2011, Santin et al. developed a new PCR protocol to amplify about 500-bp SSU rDNA fragments with high sensitivity comparable with the nested PCR, but reduced the contamination between the reactions [24]. Moreover, this fragment contains hypervariable regions that allow us to design the RFLP analysis in this study. The objective of this study is to develop a simple molecular method to differentiate all subtypes of *Blastocystis* found in humans by PCR-RFLP analysis. Although there are many studies that report subtype distribution of *Blastocystis* spp. in Thailand with the different dominant subtypes, the subtype distribution was evaluated in only selected groups in certain areas with different methods. No large-scale study in different regions was performed with the same method. Therefore, the subtype distribution of *Blastocystis* spp. in Thailand was also studied among children in all regions in Thailand in this study.

## 2. Results

### 2.1. PCR-RFPL Analysis for Differentiation of Blastocystis Subtypes 

**Figure 1:**
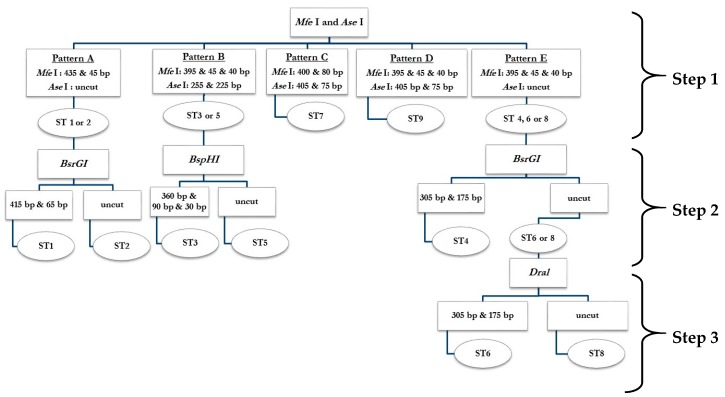
Flow chart for subtype differentiation of *Blastocystis* spp. by PCR-RFLP analysis of SSU rDNA using 3-step digestion.

**Figure 2:**
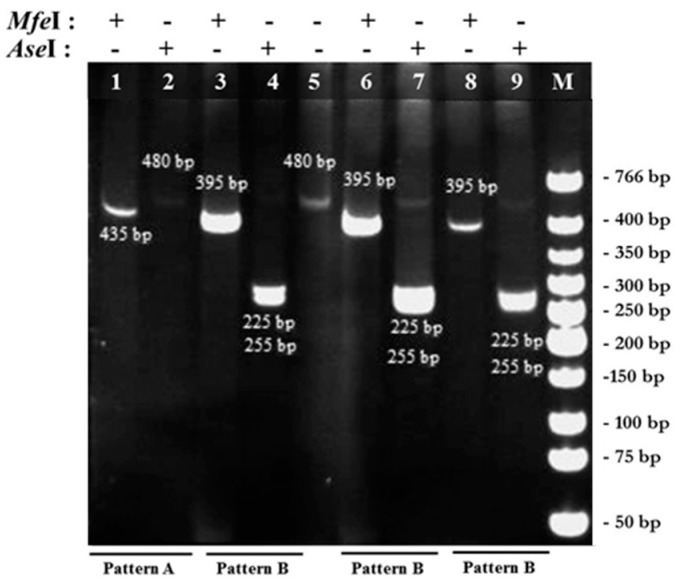
RFLP analysis of SSU rDNA by step 1 digestion with *Mfe*I and *Ase*I enzyme. Lane 1: digestion with *Mfe*I obtained fragments sized 435 bp and 45 bp; Lane 2: digestion with *Ase*I showed uncut band; Lane 3, 6 and 8: digestion with *Mfe*I obtained fragments sized 395 bp, 45 bp and 40 bp; Lane 4, 7 and 9: digestion with *Ase*I obtained fragments sized 255 bp and 225 bp; Lane 5: uncut PCR amplicons; Lane M: 1 kb DNA ladder. Lane 1, 2: Pattern A (suspected ST1 or ST2), Lane 3, 4 and 6–9: Pattern B (suspected ST3 or ST5).

**Figure 3:**
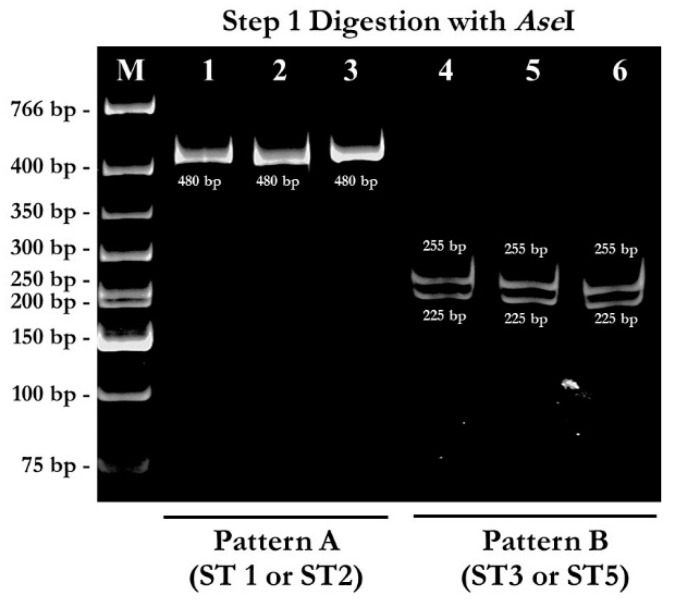
RFLP analysis of SSU rDNA by step 1 digestion with *Ase*I enzyme. Lane M: 1 kb DNA ladder; Lane 1–3: digestion with *Ase*I showed uncut bands which suspected ST1 or ST2; Lane 4–6: digestion with *Ase*I obtained fragments sized 255 bp and 225 bp which suspected ST3 or ST5.

To determine genetic diversity (subtype distribution) of *Blastocystis* spp., 480 bp of SSU rDNA fragments were successfully amplified with the Blast 505-532 (BH-SSU-F 5′ GGAGGTAGTGACAATAAATC 3′) and Blast 998-1017 (BH-SSU-R 5′ TGCTTTCGCACTTGTTCATC 3′) [24] primers from 1025 stool specimens of school-age students from 4 provinces, including Ang Thong, Nan, Nakhon Ratchasima, and Kanchanaburi which located in central, Northern, Northeastern and Western of Thailand, respectively. About 109 stool specimens were positive (26.2%) with this pair of primers. Non-specific PCR products were not found. The RFLP analysis was then used to differentiate subtypes of *Blastocystis* spp. The full diagram of PCR-RFLP analysis was shown in Figure 1. The PCR amplicons were separately digested with *Mfe* I and *Ase* I enzyme. From 109 PCR positive samples, 45 samples showed pattern A (suspected subtype 1 or subtype 2) in step 1 digestion. In pattern A, digestion with *Mfe* I enzyme showed DNA fragments sized 435 bp and 45 bp (not clearly seen) (Figure 2), while the PCR amplicons were uncut by *Ase* I enzyme (Figure 3). Out of 109 PCR positive samples, 64 samples showed pattern B (suspected subtype 3 or subtype 5) in step 1 digestion. In pattern B, digestion with *Mfe* I enzyme showed DNA fragments sized 395 bp, 45 bp, and 40 bp (not clearly seen) (Figure 2), while digestion with *Ase* I enzyme showed DNA fragments sized 255 bp, and 225 bp (Figure 2 and Figure 3). Unfortunately, pattern C, D, and E were not found in this study.

**Figure 4:**
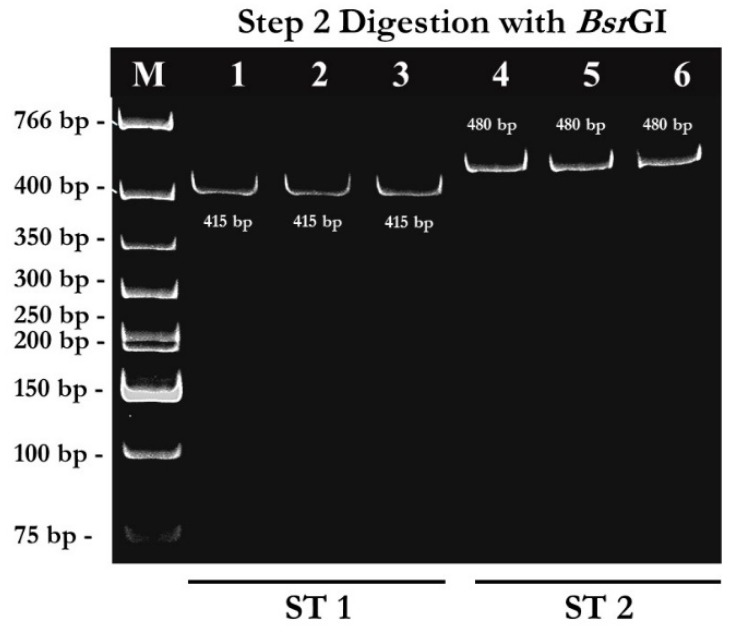
RFLP analysis of SSU rDNA by step 2 digestion with *Bsr*GI enzyme to differentiate between ST1 and ST2. Lane M: 1 kb DNA ladder; Lane 1–3: digestion with *Bsr*GI obtained fragments sized 415 bp which were identified as ST1; Lane 4–6: digestion with *Bsr*GI showed uncut bands which were identified as ST2.

From pattern A, the 480 bp-PCR amplicons were digested with *Bsr*GI enzyme in step 2 digestion to differentiate between subtype 1 and subtype 2 (Figure 1). Out of 45 samples with pattern A, 11 samples were uncut with *Bsr*GI enzyme (Figure 4). These samples were identified as subtype 2. Out of 45 samples digested with *Bsr*GI enzyme, 34 samples showed the DNA fragment sized about 415 bp (Figure 4). These samples were identified as subtype 1. 

**Figure 5:**
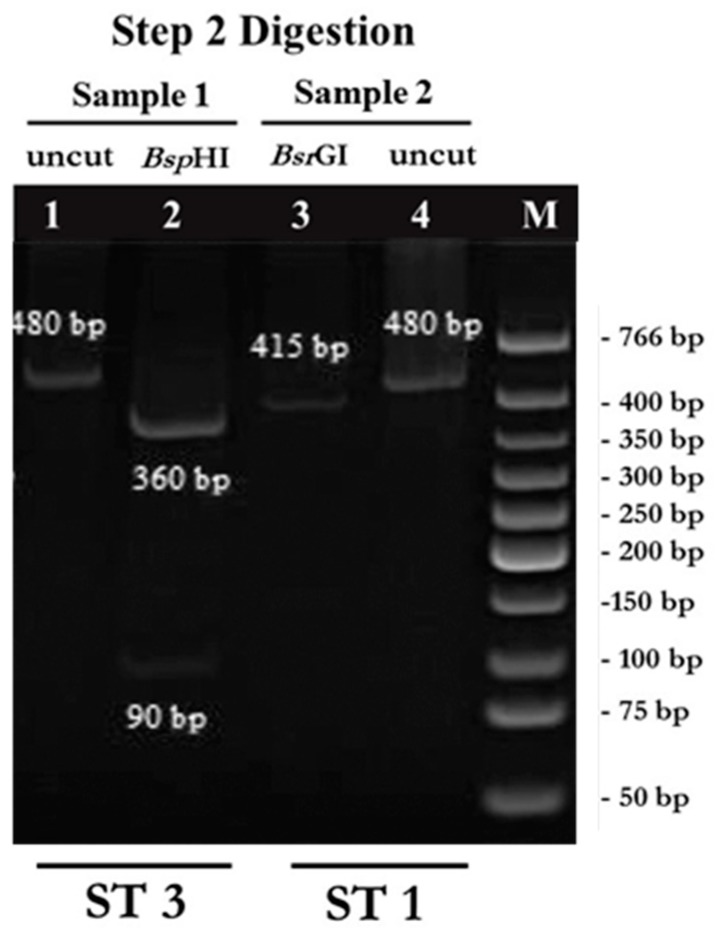
RFLP analysis of SSU rDNA by step 2 digestion with *Bsp*HI to differentiate between ST3 and ST5 and with *Bsr*GI enzymes to differentiate between ST1 and ST2. Lane M: 1 kb DNA ladder; Lane 1 and Lane 4: uncut; Lane 2: digestion with *Bsp*HI obtained fragments sized 360 and 90 bp which was identified as ST3. Lane 3: digestion with *Bsr*GI obtained fragments sized 415 bp which was identified as ST1.

From pattern B, the 480 bp-PCR amplicons were digested with *Bsp*HI in step 2 digestion to differentiate between subtype 3 and subtype 5. All of the 64 samples with pattern B in step 1 digestion showed the same pattern of RFLP analysis with *Bsp*HI enzyme. DNA fragments sized 360 bp and 90 bp were detected (Figure 5). These samples were identified as subtype 3. 

### 2.2. Phylogenetic Analysis

**Figure 6:**
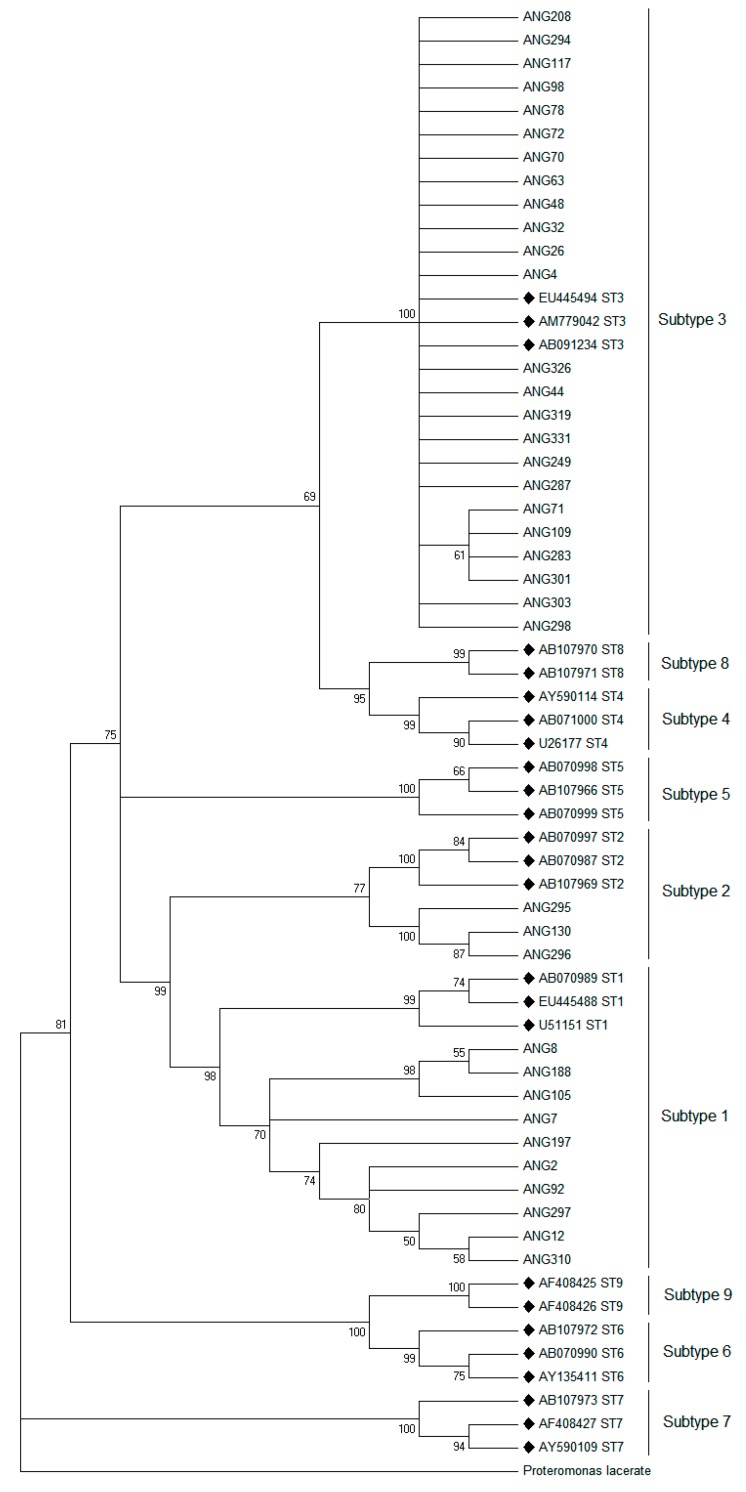
Phylogenetic tree of partial SSU-rDNA sequences of *Blastocystis* isolates, constructed using the Maximum Likelihood (ML) method. The names of reference sequences from GenBank are provided as accession numbers with black diamond (♦). *Proteromonas lacerate* (accession number U37108) is used as the out-group. Bootstrap values (%) are indicated at the internal nodes (1000 replicates). Only bootstrap values of more than 50% are shown.

After subtype differentiation with PCR-RFLP analysis, we randomly selected 37 samples with a different pattern of RFLP analysis from Ang Thong Province to perform DNA sequencing analysis. The ML tree analysis was constructed to characterize *Blastocystis* isolates in this study compared with the published sequence in the GenBank database (Table 1). The phylogenetic tree showed three distinct subtypes among *Blastocystis* spp. isolated from students in the Ang Thong province (Figure 6). These results confirmed the results of the PCR-RFLP analysis. 

### 2.3. Comparative Study of the Different Techniques for Blastocystis Detections

Out of 1025 stool samples collected from students in 4 regions of Thailand (Table 2). 416 students (40.6% prevalence) were positive for *Blastocystis* spp. by at least one method of detection (Table 3). While the highest prevalence of *Blastocystis* spp. was found among students in Kanchanaburi province (21.5%), the lowest prevalence was found among students in Nakhon Ratchasima province (0.6%) (*p* < 0.001). The prevalence in Ang Thong, and Nan province were 8.3%, and 10.2%, respectively (Table 3). 

A comparison between the direct smear, formalin-ethyl acetate concentration (FECT), Boeck and Drbohlav’s Locke-Egg-Serum (LES culture), and PCR showed a significant difference (*p* < 0.001) (Table 3). Out of 416 infected students, 105 students were positive for *Blastocystis* spp. by direct smear (25.2%), 122 students were detected by FECT (29.3%) (*p* > 0.05). Interestingly, *Blastocystis* spp. was detected in 277 samples by LES culture (66.6%). The detection rate of LES culture was higher than direct smear with statistical difference (*p* < 0.001).

However, PCR for SSU rDNA was positive in 109 samples (26.2%) (Table 3).

### 2.4. Distribution of Blastocystis Subtypes in Thai Students 

**Figure 7:**
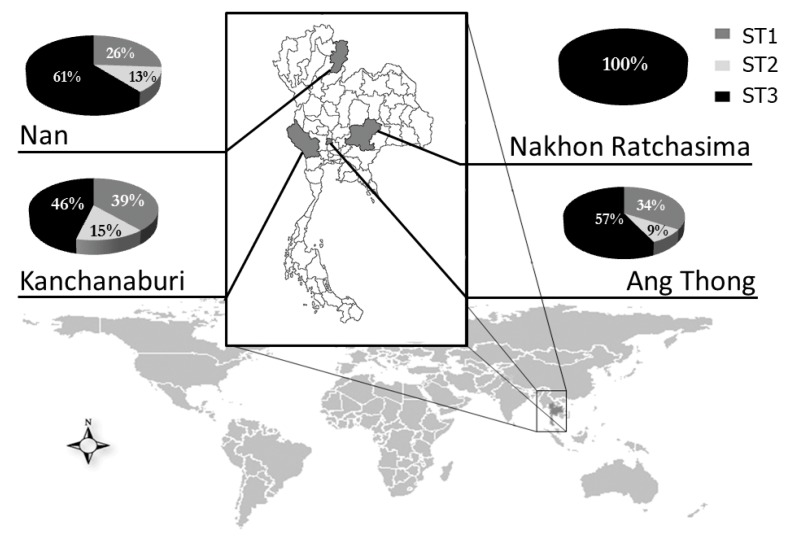
Subtype distribution of *Blastocystis* spp. in Thailand.

Using the 3-step RFLP analysis of SSU rDNA, only subtype 1-3 were found among student in Thailand. Other subtypes were not found in this population. The most common subtype was subtype 3 (64 of 109; 58.7%), followed by subtype 1 (34 of 109; 31.2%), and subtype 2 (11 of 109; 10.1%), respectively (Table 3). Subtype 3 was found with the highest prevalence in all of the province we studied (46%–100%) (Figure 7). The distribution pattern of *Blastocystis* spp. in each province was similar (subtype 3, followed by subtype 1 and subtype 2, respectively). Moreover, the proportion of each subtype in each province was not significantly different. Subtype 3 was found with the highest proportion (61%) in Nan province. Subtype 1 and subtype 2 were almost found with the highest proportion in Kanchanaburi province (39% and 15%, respectively) Among other provinces (Figure 7). Unexpectedly, we found only subtype 3 among students in Nakhon Ratchasima province, which is located in the Northeast region of Thailand (Figure 7). However, there were five positive samples in the Nakhon Ratchasima province. Therefore, this prevalence might be underestimated and did not provide an exact distribution of subtypes.

## 3. Discussion

In this study, we reported a high prevalence of *Blastocystis* spp. (40.6%) in school-age students (Table 3) from 4 provinces, which located in central, Northern, Northeastern, and Western Thailand. Our data were consistent with several studies, which show that *Blastocystis* infections are common among children in Thailand. This prevalence was higher than the prevalence in adults (14.5%–37.2%) [10,11,12] and in children in Bangkok (13.6%–31.9%) [38,52], but slightly lower than in children in orphanage (45.2%) [53]. This may be due to the overcrowded environment and poor sanitation in the orphanage. Moreover, we found that the infection rates of *Blastocystis* spp. in each region of Thailand were significantly different. The reasons for this difference may be directly related to the specific geographic characteristics, as well as to ecological, sanitary, socioeconomic, and cultural factors [54]. 

Our study showed that the highest prevalence of *Blastocystis* infection was found in Kanchanaburi (21.5%) province located in the western region of Thailand. Similar to the previous reports [55,56], the source of the infections could be drinking water contaminated with the parasites. The majority of the infected students were Karen and Myanmar migrants. Their basic health education and sanitation are poor; for examples, some families still use the cesspool latrine, while waste from the house and farm are drained directly into the nearest natural canal. They also usually drink mountain water (data from the questionnaire and observation). In this study, we found a lower prevalence rate of *Blastocystis* infection (0.49%) in the Northeastern region compared to other studies (1.6%-5.6%) [57,58]. The reasons for this difference may be due to the difference of sanitary and socioeconomic factors. The schools we surveyed in Nakhon Ratchasima province are in the urban area. The environment in schools is clean. From the questionnaire, we observed that the family status, such as occupation and estimated monthly incomes of the parents, of all students in the Nakhon Ratchasima province is quite well. About 73.1% of student’s families in Nakhon Ratchasima have mid to high monthly income (>10,000 THB; >1500 USD), while 85.8% of student’s families in Kanchanaburi have low monthly income (<10,000 THB; <1500 USD). The major occupation of parents in Nakhon Ratchasima is government officer (48.1%), while parents in Kanchanaburi is labor (70.8%). 

In the comparative study, we found that FECT could not significantly increase the detection rate of *Blastocystis* detections over direct smear (29.3%, and 25.2%, respectively) (Table 3). This might be due to the fact that some protozoa could be destroyed or distorted during the process of FECT. The highest detection rate for the detection of *Blastocystis* spp. was found by LES medium culture (66.6%). Our results showed that 37.4% of the *Blastocystis* infected patients were diagnosed only by LES medium culture, but not by direct smear and FECT. This study confirmed that detections of *Blastocystis* spp. by direct smear and FECT may not be sensitive enough for the diagnosis of the infections. PCR and real-time PCR are highly sensitive methods for the diagnosis of many infectious diseases. The sensitivity of PCR technique is 2-fold higher than the microscopic method using a modified iron-hematoxylin stain for *Blastocystis* detections [59]. However, we did not detect *Blastocystis* infection in all infected students by PCR for SSU rDNA. This could be attributed to the fact that there are many PCR inhibitors; such as bile salts, bilirubin, heme, and carbohydrates, contaminated in stool samples [60,61]. False negative results are also reported in samples with low levels of parasites [60]. Other possibilities included the limitation during the transportation of stool samples from rural areas.

The associations between the subtypes and clinical symptoms, as well as zoonosis of *Blastocystis* spp., are controversially reported. Moreover, the prevalence of each subtype reported in each country is different. Subtype 3 (41.7%–92.3%) is the most common subtype reported in most countries, followed by subtype 1 and subtype 4 [35,56]. Alternatively, the most common subtype found in Spain is subtype 4 (94.1%) [31]. Subtype 1 (51.1%) is the dominant subtype in China [62], in Libya [63], and in some regions in Thailand [10,38,55]. Moreover, the distribution pattern and the proportion of each subtype reported from each study are also different. These differences might be due to the different subtyping method of the parasites. Until now, there has been no standard genotyping method for *Blastocystis* spp. A simple molecular method for subtyping of *Blastocystis* spp. might be useful for the better understanding of the clinical association and zoonotic transmission by the parasite. 

In this study, we successfully developed a simple genotyping method to identify the subtypes of *Blastocystis* spp. in humans. We also studied the subtype distribution of *Blastocystis* spp. in Thailand. Our PCR-RFLP method is a simple, rapid, and cost-effective method, which allow us to differentiate the human *Blastocystis* subtype 1–9. However, even when we determined the subtypes of *Blastocystis* isolated from 109 stool samples collected from different regions in Thailand, we identified only subtype 1–3 among these students. The most common subtype found in all regions was subtype 3 (46%–100%) (Table 3 and Figure 7). Our results were consistent with the previous studies in Thailand that reported subtype 3 as the dominant subtype (53.2%–80%) in different populations [11,46,52,64,65]. However, our results were different to some studies that report subtype 1 as the major subtype (77.9%–100%) [10,38,55]. 

The different proportion and different pattern of subtype distribution in each region of Thailand are also reported in previous studies in Thailand. A study among students in Chachoengsao province reported that the dominant subtype is subtype 1 (77.9%), followed by subtype 2 (22.1%) [55]. *Blastocystis* spp. found in military personnel and dogs is also identified as subtype 1 [10]. Interestingly, there is no subtype 3 found among these students and military staffs. Similarly, a study in a home for girls in Bangkok, Thailand reported subtype 1 as the major subtype (94.8%), followed by subtype 6 (3.5%) and subtype 2 (1.7%) [38], however, subtype 3 was not detected in these girls. On the other hand, a study in Nonthaburi province, a province near Bangkok, Thailand reported only subtype 3 (76%), subtype 1 (16%–20%) and unidentified subtypes (4%–8%) [64]. Moreover, some studies in Thailand reported other subtypes that we did not detect in this study. Only one study reported subtype 4 (0.5%) among villagers at the Thai-Myanmar border [11]. Subtype 6 (3.5%–10%) and subtype 7 (10%–17.9%) are detected in a home for girls in Bangkok, and in a hospital in the Northeast region of Thailand [38,46,65]. These subtypes are reported as avian subtypes [23], but can transmit to humans by zoonosis [66]. Detections of zoonotic subtypes in Thailand might be due to contact with pets and farm animals [38]. Large-scaled molecular epidemiological studies, both in humans and in animals, will be useful to improve the understanding of the transmission of *Blastocystis* spp. in Thailand. Moreover, the clinical significance of *Blastocystis* in Thai patients, as well as the study of immunological aspects, should be further investigated for a better understanding of the *Blastocystis* spp. pathogenicity in humans.

## 4. Materials and Methods 

### 4.1. Population and Stool Samples 

This study was approved by the Ethics Committee of the Faculty of Medicine at Chulalongkorn University, Bangkok, Thailand (IRB No. 464/55). A total of 1025 stool samples were collected from students from 4 provinces including Ang Thong, Nan, Nakhon Ratchasima, and Kanchanaburi, which are located in central, Northern, Northeastern, and Western of Thailand, respectively. Stool collection containers were distributed to all students. To avoid contamination, all students were well informed about specific precaution and guideline for specimen collection. Written informed consent was obtained from each individual or their parents/guardians who participated in the study. 

### 4.2. Stool Examination 

The stool samples were kept under room temperature during transportation to the Department of Parasitology, Faculty of Medicine, Chulalongkorn University. To diagnose *Blastocystis* spp. stool examinations were performed. First, stool samples were examined microscopically by direct smear using in normal saline and iodine preparation. The remaining stool samples were examined by FECT, and LES medium, as previously described [9]. The LES medium culture was performed for protozoa isolation. The presence of intestinal parasite eggs, larvae, or cysts was determined microscopically. All of stool samples were independently examined by two examiners. The remaining stool samples of each individual were preserved at –20°C until DNA extraction.

### 4.3. DNA Extraction

Genomic DNA of *Blastocystis* spp. was extracted from approximately 250 mg of stool samples or from suspension collected from LES cultures using E.Z.N.A.^®^ Stool DNA Kit (OMEGA Bio-tek Inc., Norcross, GA, USA) according to the manufacturer’s protocol. DNA samples were stored at −20°C until used.

### 4.4. Polymerase Chain Reaction (PCR)

The small subunit ribosomal DNA (SSU rDNA) was amplified using the universal forward primer Blast 505-532 (BH-SSU-F 5′ GGAGGTAGTGACAATAAATC 3′) and the universal reverse primer Blast 998-1017 (BH-SSU-R 5′ TGCTTTCGCACTTGTTCATC 3′), as previously described [24]. Based on conserved regions of all available published nucleotide SSU rDNA sequences of *Blastocystis* spp. from GenBank, these primers could amplify all human *Blastocystis* subtypes. These primers produced an approximately 480 bp PCR product containing a highly variable region that allows the subtyping of *Blastocystis* spp.

The PCR was carried out in a 50 µl reaction containing 1× PCR buffer, 3 mM of MgCl_2_, 0.2 mM of each dNTP, 1U of *Taq* DNA polymerase (Thermo Scientific, MA, USA), 40 µg/mL of BSA, 0.4µM of each primer, and 200 ng of DNA template. The amplification program included the pre-heating denaturation at 95°C for 4 min, 35 cycles of denaturation at 95°C for 30 s, annealing at 50°C for 30 s, and extension at 72°C for 30 s. The final amplification cycle included an addition of a 5-minutes extension at 72°C. The aliquots of the amplicons of 480 bp were analyzed on 1% agarose gel electrophoresis in 1 × TAE buffer for 30 min. The gel was stained with ethidium bromide to visualize the DNA fragments under UV transilluminator.

### 4.5. Restriction Fragment Length Polymerase (RFLP)

To differentiate between the subtypes of *Blastocystis* spp., RFLP analysis was developed. Based on all available published nucleotide SSU rDNA sequences of *Blastocystis* subtypes from GenBank, the restriction map was predicted for all subtype using the BioEdit program version 7.0.5.2. The 3-step digestion with 5 restriction enzymes (*Mfe*I, *Ase*I, *Bsr*GI, *Bsp*HI, and *Dra*I) was designed to be used for RFLP analysis of SSU rDNA of subtype 1–9 (Figure 1). In step 1 digestion, the 480 bp-PCR amplicons were digested separately with *Mfe*I and *Ase*I enzyme (New England BioLabs, Pickering, ON, Canada). The digestion produced 5 RFLP patterns (Pattern A–E) (Figure 1). Pattern A was suspected to be subtype 1 or subtype 2. Pattern B was suspected to be subtype 3 or subtype 5. Pattern C was suspected to be subtype 7. Pattern D was suspected to be subtype 9. Pattern E was suspected to be subtype 4, or subtype 6, or subtype 8 (Figure 1). To differentiate between subtype 1 and subtype 2 (Pattern A), the 480 bp-PCR amplicons was digested with *Bsr*GI in step 2 digestion. To differentiate between subtype 3 and subtype 5 (Pattern B), the 480 bp-PCR amplicons was digested with *Bsp*HI. To differentiate subtype 4 from subtype 6 and 8 (Pattern E), the 480 bp-PCR amplicons was digested with *Bsr*GI in step 2 digestion, and then digested with *Dra*I in step 3 digestion to differentiate between subtype 6 and subtype 6 (Figure 1). 

The reaction mixture was about 6 µl of PCR product, 2 units of restriction enzyme, 1 µl of appropriate buffer and dH_2_O to final 10 µl. The RFLP mixture was incubated at 37°C for 2 h and reactivated the reaction at 65°C for 20 min. The RFLP patterns were detected on 10% acrylamide gel. The DNA fragments were stained with ethidium bromide and visualized under UV light.

### 4.6. DNA Sequencing and Phylogenetic Analysis

To confirm the subtypes of *Blastocystis*, the 20 µl of PCR products were purified by ExoSAP-IT clean-up kit (Affymetrix, High Wycombe, UK). The DNA sequencing was performed in both directions (1st Base Laboratories Sdn. Bhd., Selangor, Malaysia). All nucleotide sequences were analyzed and edited by the BioEdit Sequence Alignment Editor version 7.0.5.2. The multiple alignments with the published SSU rRNA gene available in the GenBank Database (Table 1) were performed by ClustalW multiple alignment options in the BioEdit software (Mega4 software version 4.0, Tempe, AZ, USA). Phylogenetic analysis of the aligned nucleotide sequences was also performed by MEGA software version 6. Phylogenetic trees were constructed using the Maximum Likelihood (ML) method base on the Tamura 3-parameter (T92) using a discrete Gamma distribution (+G) Tamura 3-parameter model. Branch reliability was assessed using a bootstrap analysis of 1000 replicates in the ML analysis. *Proteomonas lacetae* (U37108), closely related to *Blastocystis* spp., was used as an outgroup. 

### 4.7. Data Analysis

Data were recorded and analyzed using Microsoft Excel 2010 and GraphPad Prism version 5.01 for Windows. Association between population groups was analyzed by McNemar’s test. *P* values <0.05 were considered statistically significant.

## Figures and Tables

**Table 1:** Reference sequences of each *Blastocystis* subtype with the accession number in the GenBank database, host, as well as the country included in the analysis of this study.

Subtype	Accession Number	Country	Host	Reference
**ST1**	AB070989	Japan	Human	[47]
	EU445488	Philippines	Monkey	[39]
	U51151	USA	Human	[47]
**ST2**	AB070997	Japan	Monkey	[47]
	AB107969	Japan	Monkey	[48]
	AB070987	Japan	Human	[47]
**ST3**	AB091234	Japan	Quail	[47]
	AM779042	Turkey	Human	[49]
	EU445494	Philippines	Human	[39]
**ST4**	AB071000	Japan	Rat	[47]
	AY590114	Singapore	Rat	[27]
	U26177	USA	Guinea pig	[50]
**ST5**	AB070998	Japan	Pig	[47]
	AB070999	Japan	Pig	[47]
	AB107966	Japan	Cattle	[48]
**ST6**	AB070990	Japan	Human	[47]
	AB107972	Japan	Partridge	[48]
	AY135411	France	Turkey	[51]
**ST7**	AB107973	Japan	Goose	[48]
	AF408427	Singapore	Human	[47]
	AY590109	Singapore	Human	[27]
**ST8**	AB107970	Japan	Primate	[48]
	AB107971	Japan	Bird	[48]
**ST9**	AF408425	Japan	Human	[35]
	AF408426	Japan	Human	[35]

**Table 2:** Characteristics of total 1025 students from in 4 regions of Thailand.

Age	No.	Infected Patients
**1–6**		
Male	78 (52%)	25 (56.8%)
Female	72 (48%)	19 (43.2)
Total	150 (100%)	44 (100%)
**7–12**		
Male	96 (46.25%)	153 (47.5%)
Female	344 (53.75%)	169 (52.5%)
Total	640 (100%)	322 (100%)
**13–23**		
Male	111 (51.4%)	18 (44%)
Female	105 (48.6%)	23 (56%)
Total	216 (100%)	41 (100%)
**Unknown**	19	9
**Total**	1025	416 (40.5%)

**Table 3:** Prevalence of *Blastocystis* subtype in each province which was representative of each region in Thailand, and the comparative study of diagnosis methods of *Blastocystis* spp.

Province	N	No. of Positive	Diagnostic Method				*Blastocystis* Subtype		
			DS	FECT	LES	PCR	1	2	3
**Ang Thong**									
Male	179	43	4	12	ND	33	12	4	18
Female	151	42	5	7	ND	35	11	2	21
**Total**	330	85	9	19	ND	68	23	6	39
**Nakhon Ratchasima**									
Male	91	3	1	0	ND	2	0	0	2
Female	87	2	0	0	ND	2	0	0	2
Unknown	10	1	0	0	ND	1	0	0	1
**Total**	188	6	1	0	ND	5	0	0	5
**Nan**									
Male	115	50	21	17	39	7	3	2	2
Female	116	54	12	14	40	15	3	1	11
Unknown	9	1	0	1	0	1	0	0	1
**Total**	240	105	33	32	79	23	6	3	14
**Kanchanaburi**									
Male	122	100	34	31	89	6	2	1	3
Female	138	113	27	37	103	7	3	1	3
Unknown	7	7	1	3	6	0	0	0	0
**Total**	267	220	62	71	198	13	5	2	6
**Total**	1025	416	105	122	277	109	34	11	64
		(40.6%)	(25.2%)	(29.3%)	(66.6%) *	(26.2%)	(31.2%)	(10.1%)	(58.7%)

DS: Direct smear; FECT: formalin ethyl acetate concentration; LES: Boeck and Drbohlav’s Locke-Egg Serum medium culture; PCR: polymerase chain reaction; *: Values is significantly different from DS (*p* < 0.001) by McNemar’s test.

## References

[1] Garavelli P.L., Scaglione L., Bicocchi R., Libanore M. (1990). Blastocystosis: A new disease in the acquired immunodeficiency syndrome?. Int. J. STD AIDS.

[2] Segui R., Klisiowicz D., Oishi C.Y., Toledo R., Esteban J.G., Munoz-Antoli C. (2017). Intestinal symptoms and Blastocystis load in schoolchildren of Paranagua Bay, Parana, Brazil. Rev. Inst. Med. Trop. Sao Paulo.

[3] Roberts T., Stark D., Harkness J., Ellis J. (2014). Update on the pathogenic potential and treatment options for Blastocystis sp.. Gut Pathog..

[4] Hirata T., Nakamura H., Kinjo N., Hokama A., Kinjo F., Yamane N., Fujita J. (2007). Prevalence of Blastocystis hominis and Strongyloides stercoralis infection in Okinawa, Japan. Parasitol. Res..

[5] Amin O.M. (2002). Seasonal prevalence of intestinal parasites in the United States during 2000. Am. J. Trop. Med. Hyg..

[6] Pegelow K., Gross R., Pietrzik K., Lukito W., Richards A.L., Fryauff D.J. (1997). Parasitological and nutritional situation of school children in the Sukaraja district, West Java, Indonesia. Southeast Asian J. Trop. Med. Public Health.

[7] Rayan H.Z., Ismail O.A., El Gayar E.K. (2007). Prevalence and clinical features of Dientamoeba fragilis infections in patients suspected to have intestinal parasitic infection. J. Egypt Soc. Parasitol..

[8] El Safadi D., Gaayeb L., Meloni D., Cian A., Poirier P., Wawrzyniak I., Delbac F., Dabboussi F., Delhaes L., Seck M. (2014). Children of Senegal River Basin show the highest prevalence of Blastocystis sp. ever observed worldwide. BMC Infect. Dis..

[9] Nuchprayoon S., Sanprasert V., Kaewzaithim S., Saksirisampant W. (2009). Screening for intestinal parasitic infections among Myanmar migrant workers in Thai food industry: A high-risk transmission. J. Immigr. Minor. Health.

[10] Leelayoova S., Siripattanapipong S., Naaglor T., Taamasri P., Mungthin M. (2009). Prevalence of intestinal parasitic infections in military personnel and military dogs, Thailand. J. Med. Assoc. Thail..

[11] Popruk S., Udonsom R., Koompapong K., Mahittikorn A., Kusolsuk T., Ruangsittichai J., Palasuwan A. (2015). Subtype distribution of Blastocystis in Thai-Myanmar border, Thailand. Korean J. Parasitol..

[12] Yowang A., Tsaousis A.D., Chumphonsuk T., Thongsin N., Kullawong N., Popluechai S., Gentekaki E. (2018). High diversity of Blastocystis subtypes isolated from asymptomatic adults living in Chiang Rai, Thailand. Infect. Genet. Evol. J. Mol. Epidemiol. Evol. Genet. Infect. Dis..

[13] Udonsom R., Prasertbun R., Mahittikorn A., Mori H., Changbunjong T., Komalamisra C., Pintong A.R., Sukthana Y., Popruk S. (2018). Blastocystis infection and subtype distribution in humans, cattle, goats, and pigs in central and western Thailand. Infect. Genet. Evol. J. Mol. Epidemiol. Evol. Genet. Infect. Dis..

[14] Amin O.M. (2006). Epidemiology of Blastocystis hominis in the United States. Res. J. Parasitol..

[15] Armentia A., Mendez J., Gomez A., Sanchis E., Fernandez A., de la Fuente R., Sanchez P. (1993). Urticaria by Blastocystis hominis. Successful treatment with paromomycin. Allergol. Immunopathol. (Madr.).

[16] Gupta R., Parsi K. (2006). Chronic urticaria due to Blastocystis hominis. Australas. J. Dermatol..

[17] Katsarou-Katsari A., Vassalos C.M., Tzanetou K., Spanakos G., Papadopoulou C., Vakalis N. (2008). Acute urticaria associated with amoeboid forms of Blastocystis sp. subtype 3. Acta Derm. Venereol..

[18] Tan T.C., Suresh K.G. (2006). Predominance of amoeboid forms of Blastocystis hominis in isolates from symptomatic patients. Parasitol. Res..

[19] Hussein E.M., Hussein A.M., Eida M.M., Atwa M.M. (2008). Pathophysiological variability of different genotypes of human Blastocystis hominis Egyptian isolates in experimentally infected rats. Parasitol. Res..

[20] Olivo-Diaz A., Romero-Valdovinos M., Gudino-Ramirez A., Reyes-Gordillo J., Jimenez-Gonzalez D.E., Ramirez-Miranda M.E., Martinez-Flores W.A., Martinez-Hernandez F., Flisser A., Maravilla P. (2012). Findings related to IL-8 and IL-10 gene polymorphisms in a Mexican patient population with irritable bowel syndrome infected with Blastocystis. Parasitol. Res..

[21] Clark C.G. (1997). Extensive genetic diversity in Blastocystis hominis. Mol. Biochem. Parasitol..

[22] Alfellani M.A., Taner-Mulla D., Jacob A.S., Imeede C.A., Yoshikawa H., Stensvold C.R., Clark C.G. (2013). Genetic diversity of blastocystis in livestock and zoo animals. Protist.

[23] Stensvold C.R., Alfellani M.A., Nørskov-Lauritsen S., Prip K., Victory E.L., Maddox C., Nielsen H.V., Clark C.G. (2009). Subtype distribution of Blastocystis isolates from synanthropic and zoo animals and identification of a new subtype. Int. J. Parasitol..

[24] Santin M., Gomez-Munoz M.T., Solano-Aguilar G., Fayer R. (2011). Development of a new PCR protocol to detect and subtype Blastocystis spp. from humans and animals. Parasitol. Res..

[25] Fayer R., Elsasser T., Gould R., Solano G., Urban J., Santin M. (2014). Blastocystis tropism in the pig intestine. Parasitol. Res..

[26] Parkar U., Traub R.J., Vitali S., Elliot A., Levecke B., Robertson I., Geurden T., Steele J., Drake B., Thompson R.C.A. (2010). Molecular characterization of Blastocystis isolates from zoo animals and their animal-keepers. Vet. Parasitol..

[27] Noël C., Dufernez F., Gerbod D., Edgcomb V.P., Delgado-Viscogliosi P., Ho L.C., Singh M., Wintjens R., Sogin M.L., Capron M. (2005). Molecular phylogenies of Blastocystis isolates from different hosts: Implications for genetic diversity, identification of species, and zoonosis. J. Clin. Microbiol..

[28] Sanpool O., Laymanivong S., Thanchomnang T., Rodpai R., Sadaow L., Phosuk I., Maleewong W., Intapan P.M. (2017). Subtype identification of human Blastocystis spp. isolated from Lao People’s Democratic Republic. Acta Trop..

[29] Stensvold C.R., Arendrup M.C., Nielsen H.V., Bada A., Thorsen S. (2008). Symptomatic infection with Blastocystis sp. subtype 8 successfully treated with trimethoprim-sulfamethoxazole. Ann. Trop. Med. Parasitol..

[30] Roberts T., Stark D., Harkness J., Ellis J. (2013). Subtype distribution of Blastocystis isolates identified in a Sydney population and pathogenic potential of Blastocystis. Eur. J. Clin. Microbiol. Infect. Dis..

[31] Dominguez-Marquez M.V., Guna R., Munoz C., Gomez-Munoz M.T., Borras R. (2009). High prevalence of subtype 4 among isolates of Blastocystis hominis from symptomatic patients of a health district of Valencia (Spain). Parasitol. Res..

[32] Dogruman-Al F., Dagci H., Yoshikawa H., Kurt O., Demirel M. (2008). A possible link between subtype 2 and asymptomatic infections of Blastocystis hominis. Parasitol. Res..

[33] Yoshikawa H., Nagano I., Wu Z., Yap E.H., Singh M., Takahashi Y. (1998). Genomic polymorphism among Blastocystis hominis strains and development of subtype-specific diagnostic primers. Mol. Cell. Probes.

[34] Stensvold C.R. (2013). Comparison of sequencing (barcode region) and sequence-tagged-site PCR for Blastocystis subtyping. J. Clin. Microbiol..

[35] Yoshikawa H., Wu Z., Kimata I., Iseki M., Ali I.K.M.D., Hossain M.B., Zaman V., Haque R., Takahashi Y. (2004). Polymerase chain reaction-based genotype classification among human Blastocystis hominis populations isolated from different countries. Parasitol. Res..

[36] Stensvold C.R., Alfellani M., Clark C.G. (2012). Levels of genetic diversity vary dramatically between Blastocystis subtypes. Infect. Genet. Evol. J. Mol. Epidemiol. Evol. Genet. Infect. Dis..

[37] Scanlan P.D., Stensvold C.R., Cotter P.D. (2015). Development and Application of a Blastocystis Subtype-Specific PCR Assay Reveals that Mixed-Subtype Infections Are Common in a Healthy Human Population. Appl. Environ. Microbiol..

[38] Thathaisong U., Siripattanapipong S., Mungthin M., Pipatsatitpong D., Tan-ariya P., Naaglor T., Leelayoova S. (2013). Identification of Blastocystis subtype 1 variants in the Home for Girls, Bangkok, Thailand. Am. J. Trop. Med. Hyg..

[39] Rivera W.L. (2008). Phylogenetic analysis of Blastocystis isolates from animal and human hosts in the Philippines. Vet. Parasitol..

[40] Parkar U., Traub R.J., Kumar S., Mungthin M., Vitali S., Leelayoova S., Morris K., Thompson R.C. (2007). Direct characterization of Blastocystis from faeces by PCR and evidence of zoonotic potential. Parasitology.

[41] Termmathurapoj S., Leelayoova S., Aimpun P., Thathaisong U., Nimmanon T., Taamasri P., Mungthin M. (2004). The usefulness of short-term in vitro cultivation for the detection and molecular study of Blastocystis hominis in stool specimens. Parasitol. Res..

[42] Init I., Foead A.L., Fong M.Y., Yamazaki H., Rohela M., Yong H.S., Mak J.W. (2007). Restriction enzyme digestion analysis of PCR-amplified DNA of Blastocystis hominis isolates. Southeast Asian J. Trop. Med. Public Health.

[43] Hoevers J., Holman P., Logan K., Hommel M., Ashford R., Snowden K. (2000). Restriction-fragment-length polymorphism analysis of small-subunit rRNA genes of Blastocystis hominis isolates from geographically diverse human hosts. Parasitol. Res..

[44] Abe N., Wu Z., Yoshikawa H. (2003). Molecular characterization of Blastocystis isolates from birds by PCR with diagnostic primers and restriction fragment length polymorphism analysis of the small subunit ribosomal RNA gene. Parasitol. Res..

[45] Abe N., Wu Z., Yoshikawa H. (2003). Zoonotic genotypes of Blastocystis hominis detected in cattle and pigs by PCR with diagnostic primers and restriction fragment length polymorphism analysis of the small subunit ribosomal RNA gene. Parasitol. Res..

[46] Jantermtor S., Pinlaor P., Sawadpanich K., Pinlaor S., Sangka A., Wilailuckana C., Wongsena W., Yoshikawa H. (2013). Subtype identification of Blastocystis spp. isolated from patients in a major hospital in northeastern Thailand. Parasitol. Res..

[47] Arisue N., Hashimoto T., Yoshikawa H. (2003). Sequence heterogeneity of the small subunit ribosomal RNA genes among blastocystis isolates. Parasitology.

[48] Abe N. (2004). Molecular and phylogenetic analysis of Blastocystis isolates from various hosts. Vet. Parasitol..

[49] Ozyurt M., Kurt O., Molbak K., Nielsen H.V., Haznedaroglu T., Stensvold C.R. (2008). Molecular epidemiology of Blastocystis infections in Turkey. Parasitol. Int..

[50] Leipe D.D., Tong S.M., Goggin C.L., Slemenda S.B., Pieniazek N.J., Sogin M.L. (1996). 16S-like rDNA sequences from Developayella elegans, Labyrinthuloides haliotidis, and Proteromonas lacertae confirm that the stramenopiles are a primarily heterotrophic group. Eur. J. Protistol..

[51] Noel C., Peyronnet C., Gerbod D., Edgcomb V.P., Delgado-Viscogliosi P., Sogin M.L., Capron M., Viscogliosi E., Zenner L. (2003). Phylogenetic analysis of Blastocystis isolates from different hosts based on the comparison of small-subunit rRNA gene sequences. Mol. Biochem. Parasitol..

[52] Pipatsatitpong D., Leelayoova S., Mungthin M., Aunpad R., Naaglor T., Rangsin R. (2015). Prevalence and Risk Factors for Blastocystis Infection among Children and Caregivers in a Child Care Center, Bangkok, Thailand. Am. J. Trop. Med. Hyg..

[53] Saksirisampant W., Nuchprayoon S., Wiwanitkit V., Yenthakam S., Ampavasiri A. (2003). Intestinal parasitic infestations among children in an orphanage in Pathum Thani province. J. Med. Assoc. Thail..

[54] Soriano S.V., Barbieri L.M., Pierangeli N.B., Giayetto A.L., Manacorda A.M., Castronovo E., Pezzani B.C., Minvielle M., Basualdo J.A. (2001). Intestinal parasites and the environment: Frequency of intestinal parasites in children of Neuquen, Patagonia, Argentina. Rev. Latinoam. Microbiol..

[55] Leelayoova S., Siripattanapipong S., Thathaisong U., Naaglor T., Taamasri P., Piyaraj P., Mungthin M. (2008). Drinking water: A possible source of blastocystis spp. subtype 1 infection in schoolchildren of a rural community in central Thailand. Am. J. Trop. Med. Hyg..

[56] Li L.H., Zhou X.N., Du Z.W., Wang X.Z., Wang L.B., Jiang J.Y., Yoshikawa H., Steinmann P., Utzinger J., Wu Z. (2007). Molecular epidemiology of human Blastocystis in a village in Yunnan province, China. Parasitol. Int..

[57] Kitvatanachai S., Boonslip S., Watanasatitarpa S. (2008). Intestinal parasitic infections in Srimum suburban area of Nakhon Ratchasima Province, Thailand. Trop. Biomed..

[58] Boonjaraspinyo S., Boonmars T., Kaewsamut B., Ekobol N., Laummaunwai P., Aukkanimart R., Wonkchalee N., Juasook A., Sriraj P. (2013). A cross-sectional study on intestinal parasitic infections in rural communities, northeast Thailand. Korean J. Parasitol..

[59] Roberts T., Barratt J., Harkness J., Ellis J., Stark D. (2011). Comparison of microscopy, culture, and conventional polymerase chain reaction for detection of blastocystis sp. in clinical stool samples. Am. J. Trop. Med. Hyg..

[60] Forsell J., Koskiniemi S., Hedberg I., Edebro H., Evengard B., Granlund M. (2015). Evaluation of factors affecting real-time PCR performance for diagnosis of Entamoeba histolytica and Entamoeba dispar in clinical stool samples. J. Med. Microbiol..

[61] Oikarinen S., Tauriainen S., Viskari H., Simell O., Knip M., Virtanen S., Hyoty H. (2009). PCR inhibition in stool samples in relation to age of infants. J. Clin. Virol. Off. Publ. Pan Am. Soc. Clin. Virol..

[62] Yan Y., Su S., Lai R., Liao H., Ye J., Li X., Luo X., Chen G. (2006). Genetic variability of Blastocystis hominis isolates in China. Parasitol. Res..

[63] Abdulsalam A.M., Ithoi I., Al-Mekhlafi H.M., Al-Mekhlafi A.M., Ahmed A., Surin J. (2013). Subtype distribution of Blastocystis isolates in Sebha, Libya. PLoS ONE.

[64] Boondit J., Pipatsatitpong D., Mungthin M., Taamasri P., Tan-ariya P., Naaglor T., Leelayoova S. (2014). Incidence and risk factors of blastocystis infection in orphans at the Babies’ Home, Nonthaburi Province, Thailand. J. Med. Assoc. Thail..

[65] Sanpool O., Laoraksawong P., Janwan P., Intapan P.M., Sawanyawisuth K., Thanchomnang T., Changtrakul Y., Maleewong W. (2015). Genetic Subtypes of Blastocystis Isolated from Thai Hospitalized Patients in Northeastern Thailand. Southeast Asian J. Trop. Med. Public Health.

[66] Yoshikawa H., Abe N., Wu Z. (2004). PCR-based identification of zoonotic isolates of Blastocystis from mammals and birds. Microbiology.

